# A Niemann‐pick C1 disease child with BCG-itis: a case report and analysis

**DOI:** 10.1186/s12887-021-02671-7

**Published:** 2021-05-04

**Authors:** Jing-jing Lin, Xu-hui Liu, Lu Xia, Yan-ling Feng, Xiu-hong Xi, Shui-hua Lu

**Affiliations:** Shanghai Public Health Clinical Center, Fudan University, Shanghai, China

**Keywords:** Niemann‐pick C1 disease, BCG-itis, Case report

## Abstract

**Background:**

Niemann-Pick C disease is a rare autosomal recessive lysosomal lipid storage disorder. Some primary immunodeficiency diseases patients developed regional disease or disseminated disease after vaccinating BCG. It is unclear whether NPC gene deficiency is associated with Mycobacteria infection.

**Case presentation:**

We report and discuss a case of a child who presented at the age of 6 months with NPC1 and BCG-itis. The patient was treated with Miglustat and the symptom of lymphadenopathy was improved.

**Conclusions:**

We reasonably speculate that NPC1 is a susceptibility gene of *Mtb* infection and mainly affects innate immunity. Once diagnosed, the infant should not be vaccinated with BCG and early treated.

## Background

Niemann-Pick C (NPC) disease is a rare autosomal recessive lysosomal lipid storage disorder caused by mutations of either NPC1 or NPC2 gene. It is estimated to affect 1 in 150,000 live births [1]. 95 % NPC disease was associated with NPC1 mutation, with abnormal accumulation of unesterified cholesterol (UC) and glycolipids in late endosomes (LE) and lysosomes (LY) [2]. Characterized by forming lipid-laden foam cells, macrophages are also called Niemann-Pick cells (NPC cells), which appear in the organs of the monocyte-macrophage system [3], such as the liver and the spleen, and also appear in the central nervous system and the lung.

It is unclear whether this congenital gene deficiency is associated with Mycobacteria infection. Here we present a unique case with congenital NPC1 mutation and was infected by Bacillus Calmette Guerin strain after vaccination. This case report raised a suspicion that the NPC1 mutation is probably associated with host susceptibility to Mycobacteria.

## Case presentation

**Fig. 1:**
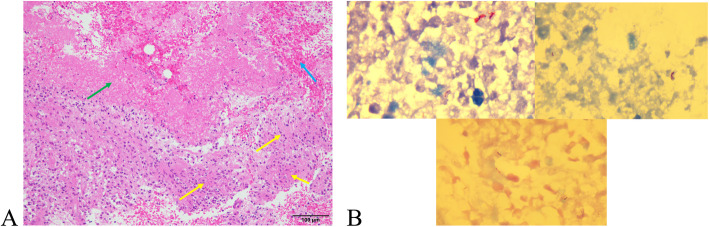
**a** Histopathology of the lymph nodule on the left supraclavicular showing granuloma (yellow arrow), coagulative necrosis (green arrow) with inflammatory cell infiltration, and focal hemorrhage (blue arrow) complete coagulation necrosis (HE x 200). **b** Acid-fast staining of the histopathology showing positive (red)

A six months and twelve days old girl presented with left supraclavicular and left axillary abscesses without fever, night sweats, weight loss, growth retardation, or other visible abnormality. A visible mass was detected 4 months after BCG vaccination, and progressed and turned suppurative in one month. The BCG was administrated according to domestic law. No scar was observed at the injection site. A biopsy was performed before visiting our site and showed chronic granulomatous inflammation without a sign of malignancy. We performed another biopsy (Fig. 1) and the aspirate was examined by the histopathological method and microbiological tests including bacteria cultured, acid-fast staining, nucleic acid test (Gene Xpert MTB/RIF assay and PCR). The reports showed positive for the acid-fast staining and the Gene Xpert MTB/RIF assay. The PCR identification showed *M.bovis* BCG. The Roche solid culture and BACTEC MGITTM960 culture were also positive. The MPB64 assay and T-SPOT.TB assay were negative. Ultrasonography was advised and showed us that splenomegaly, left supraclavicular and axillary lymphadenopathy, bilateral cervical and submandibular lymphadenopathy (smaller), and other regions were normal. Chest CT and brain MRI showed normal. Genetic testing was also advised and showed that there are two heterozygous mutations in the NPC1 exon region ((NM_000271.4) c.178 C > T, c.2728G > A) (Table 1) that may cause NPC1. To verify, PCR amplification of NPC1 locus and Sanger sequencing were performed, and the result was consistent with previous.

**Table 1:** Gene locus((NM_000271.4) c.178 C > T, c.2728G > A) of the gene NPC1 was amplified by PCR and identified by Sanger sequencing

	Variance region	Base(s) change
**Patient**	E2	c.178 C > T (p.Q60X)
	E18	c.2728G > A (p.G910S)
**Patient’s father**	E2	c.178 C > T (p.Q60X)
**Patient’s mother**	E18	c.178 C > T (p.Q60X)

**Fig. 2:**
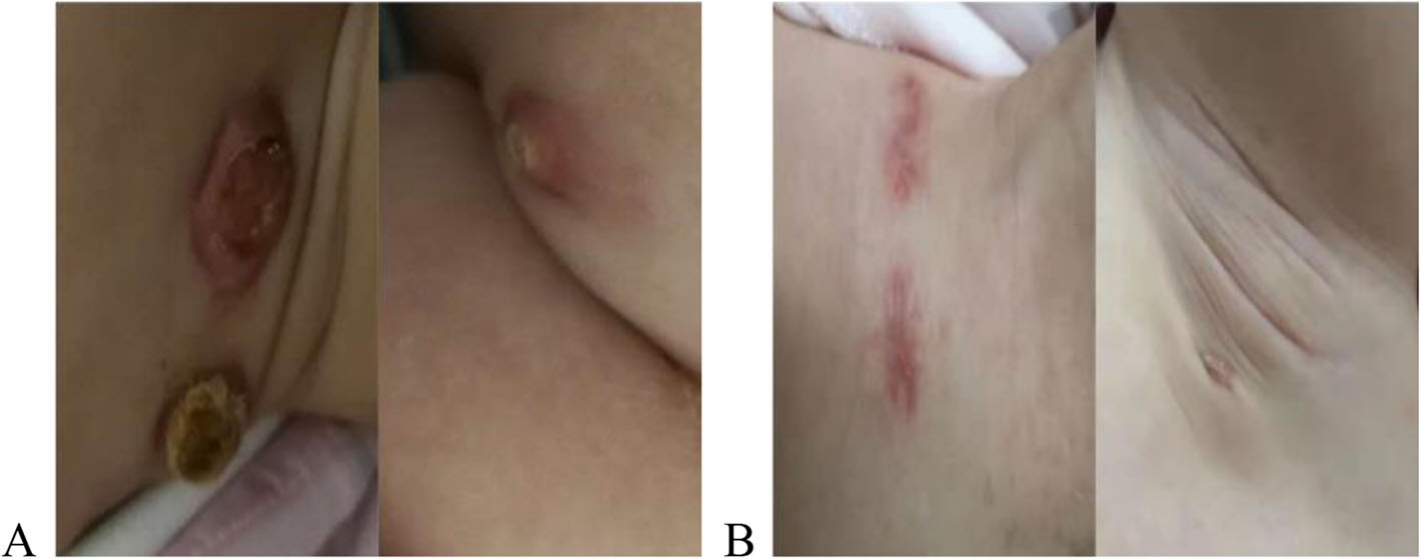
**a** 1 week after the biopsy. **b** 10 months after therapy. Left supraclavicular (left) and left axillary lymph nodes (right

Because splenomegaly may result from NPC1 and the patient is treated NPC1 by Miglustat, an iminosugar inhibitor of glucosylceramide synthase, which approved in Europe and in a number of other countries for the treatment of neurological manifestations of NPC, BCG-itis was our first diagnosis and the immunomodulator was our preferred treatment rather than anti-tuberculosis. After 10 months of return visits, the enlarged lymph nodes became smaller gradually (Fig. 2).

## Discussion and conclusion

BCG, an attenuated form of *M. bovis*, is the only vaccination approved to apply to clinic. WHO recommends BCG vaccinates newborns to prevent from miliary and meningeal forms of TB in high TB burden countries. Some primary immunodeficiency diseases (PIDs) make patients vulnerable to weakly virulent pathogens, including BCG, although BCG has good safety. Hence, some children suffering from PIDs developed regional disease (BCG-itis) or disseminated disease (BCG-osis) after vaccinating BCG [4]. BCG-osis usually cause distant lymph nodes, liver and spleen enlargement, bone also involved. BCG-itis is characterized as purulent regional lymphadenitis, local erythema accompanied by ipsilateral regional lymph node enlargement. Hence, the diagnosis of them is based on the history of BCG vaccination, clinical, laboratory and imaging characters [5]. Differ from BCG-osis, BCG-itis may not require anti-tuberculosis therapy.

One week after first visiting our outpatient, we got the result of T-SPOT, smear, Gene-Xpert and PCR of the aspirated material, and based on chest CT, brain MRI, BCG-itis was strongly suspected, we offer immunomodulator therapy instead of anti-tuberculosis. A few weeks later, the result of culture and bacteria identification confirmed our diagnosis.

NPC is commonly considered as a neurovisceral disease [6], and mainly manifest as a progressive neurodegenerative disorder, consisting of cerebellar ataxia, seizures, progressive dementia, vertical supranuclear gaze palsy, dysarthria and dysphagia, and neonatal cholestatic jaundice, hepatosplenomegaly [2] and recurrent respiratory infections [7] are other common features. Each manifestation follows independent courses and occurs at different times. Systemic disease may be absent in 15 % patients [8], always precedes neurological symptoms. Another clinical feature is the age of onset ranging from the perinatal period to more than 70 years old [6]. The diagnosis of NPC is according to laboratory test——the skin fibroblasts demonstrate the impaired ability to transport intracellular cholesterol [8]. Miglustat has been approved for NPC by the European Union, which can postpone disease progress in late-onset and not too advanced patients, especially patients who are at late-onset of disease [6]. NPC1 of the patient is diagnosed based on twice gene testing results and the clinical presentation of splenomegaly. And Miglustat was used to treat NPC1 under the guidance of a specialist as soon as diagnosed.

The main transport pathway of intracellular cholesterol can be described simply as following: Low-density lipoprotein (LDL) recognizes, aggregates and binds to the LDL receptor on the cell membrane, then enters cells via endocytosis. After endosome fuses with the lysosome, the cholesterol ester is disassembled into free UC. The protein NPC2, a soluble, cholesterol-binding luminal protein in the lumen, transfers cholesterol to the N-terminal domain of NPC 1, a transmembrane glycoprotein in the LE/LY. Then, the protein NPC1 transfers UC to organelle (e.g. ER) [2, 9]. That is a crucial pathway of intracellular cholesterol trafficking. However, the mutation of lysosomal integral membrane protein NPC1 [10] makes lips cannot be transferred by the protein NPC1 and UC, glycosphingolipids, sphingomyelin, and sphingosine accumulating in cells [1]. Moreover, cholesterol is a beneficial factor of *Mtb* intracellular existing persistently. Taken together, it is reasonable to believe that NPC cells are beneficial for *Mtb* survival.

The immune response, critical for control of *Mtb* infection in humans, starts with macrophages. After infection, macrophages phagocytize *Mtb* and become phagosomes to fused with lysosomes, then form phagolysosomes. The acid hydrolase in lysosomes kills *Mtb* or inhibits its growth (phagosome acidification). Macrophages can also kill *Mtb* by generating free radicals, autophagy, and initiating adaptive immune responses as antigen-presenting cells [11]. Studies have shown that the phagocytosis of macrophages and the long-term survival of intracellular *Mtb* depend on cholesterol. Accumulation of cholesterol is not only utilized by *Mtb* as a source of carbon for survival but also decreases the PH of lysosome to support the survival of *Mtb *[12, 13]. Moreover, it is a consistent and prominent feature that lipid-laden macrophages appear in both granulomatous lesions and *Mtb* infectious lesions [14]. It can be speculated that the bactericidal activity of NPC cells decreases and the viability of intracellular *Mtb* increases. NK cells are also involved in innate immunity. NK cells inhibit *Mtb* growth directly via cytotoxic mechanisms and indirectly via immune-stimulating macrophages active [11]. A study of a mouse model found that NPC1^−/−^ mouse is deficient in the peripheral immune system, especially NK cells. The frequency of NK cells decreases in circulation and the function is weaker [10]. NKT cells were also revealed involved in response to *Mtb* infection [15], and iNKT cell control of *Mtb* growth was CD1d-dependent [16], which presented lipid antigens. The NPC cells might affect the antigen presentation pathway, and restrict iNKT cell effector function during infection.

As mentioned above, phagosome acidification is one mechanism of anti-tuberculosis innate immunity. In 2012, research demonstrated that all-trans retinoic acid (ATRA)-induced cellular antimicrobial activity depended on the expression and function of the protein NPC2. In detail, ATRA induces the protein NPC2 expressing to decrease intracellular cholesterol and increase in lysosome acidification. Conversely, ATRA-induced antimicrobial activity can be ablated because of the loss of the protein NPC2 [13]. In the cholesterol transport pathway, the protein NPC1 locates downstream of the protein NPC2, and both of them play a role in transporting UC. Taken together, it is reasonable to speculate that the non-expression of NPC1 can cause a decrease in lysosomal acidification, which affects a key process in antimicrobial activity against *Mtb* of innate immunity.

LDL can also be oxidized to oxidized LDL (oxLDL) which can be phagocytized by macrophages and vascular endothelial cells. It has been demonstrated that lysosomal cholesterol esterase cannot disassemble oxLDL-derived lipids, which leads to lipid accumulation in lysosomes in macrophages, and dysfunction in intracellular cholesterol transportation and effluxion [12]. This is similar to what happens in NPC cells. Another experiment showed that cholesterol accumulation due to oxLDL uptake or NPC1 deficiency results in lysosomal dysfunction in macrophages by interfering with phagolysosomes trafficking, maturation, and fusion; inhibiting autophagy; increasing the PH of lysosomal; damaging the lysosomal membrane directly and triggering downstream inflammatory [12], which is beneficial for the growth of *Mtb*, as some mechanisms consistent with immune evasion mechanism of *Mtb*.

Besides, a study indicates that foamy macrophages containing oxLDL, appearing during TB development, support *Mtb* survival because of intracellular cholesterol accumulation, which is considered significant for the development of tuberculous granulomas and persistence of *Mtb* infection [12].

Besides, a study indicated that the phenotype of cells infected with *M. bovis* BCG and wild-type Mtb is consistent with NPC cells [17], and the protein NPC1 can regulate liver X receptor (LXR)-dependent cholesterol efflux and relieve cholesterol-induced oxidative stress in macrophages [9]. Based on that, it is reasonable speculation that the protein NPC1 is an effective factor to reduce the viability and even prevent infection of *M. bovis* BCG and *Mtb*.

The live attenuated vaccine BCG is safe for the general population but risky in patients with PIDs, especially for those with BCG-osis. As NPC1 is a rare gene deficiency disease, there is no evident result linking NPC1 with BCG-itis. However, there is a lot of evidence relating NPC1 with *Mtb* that NPC cells are beneficial for *Mtb* infection and intracellular survival. Moreover, Crohn’s disease has been reported in association with NPC, Schwerd et al. [18] demonstrated that NPC1 mutations in vitro induce a defect in autophagy which leads to impaired NOD2-mediated bacterial killing by macrophages. In summary, we can reasonably speculate that NPC1 is a susceptibility gene of *Mtb* infection and mainly affects innate immunity. Therefore, mutation of NPC1 may cause adverse reactions after BCG vaccination, but it tends to result in BCG-itis. NPC1 is an autosomal recessive disorder, the clinical symptoms appear when the gene is homozygous (NPC1^−/−^). Therefore, it is recommended that genetic counseling in patients with NPC1 in the family, antenatal tests (e.g. amniocentesis) to clarify whether there is a defect in the NPC1 gene. Once diagnosed, the infant should not be vaccinated with BCG and early treated. But in order to determine the relationship between NPC1 with *M. bovis* BCG, it is necessary to experiment in animals and search for more NPC1 patients with BCG-itis.

## Data Availability

All data generated or analyzed during this study are included in this published article. Data sharing does not apply to this report as no data sets were generated or analyzed.

## Abbreviations

NPC: Niemann-Pick C
UC: Unesterified cholesterol
LE: Late endosomes
LY: Lysosomes
NPC cells: Niemann-Pick cells
PIDs: Primary immunodeficiency diseases
LDL: Low-density lipoprotein
ATRA: All-trans retinoic acid
oxLDL: Oxidized LDL
LXR: Liver X receptor

## References

[1] Yu X-H, Jiang N, Yao P-B, Zheng X-L, Cayabyab FS, Tang C-K (2014). NPC1, intracellular cholesterol trafficking and atherosclerosis. Clin Chim Acta.

[2] Tseng W-C, Loeb HE, Pei W (2018). Modeling Niemann-Pick disease type C1 in zebrafish: a robust platform forin vivo screening of candidate therapeutic compounds. Disease Models Mechanisms.

[3] Guillemot N, Troadec C, de Villemeur TB, Clément A, Fauroux B (2007). Lung disease in niemann–pick disease. Pediatr Pulmonol.

[4] Norouzi S, Aghamohammadi A, Mamishi S, Rosenzweig SD, Rezaei N (2012). Bacillus Calmette-Guerin (BCG) complications associated with primary immunodeficiency diseases. J Infect.

[5] Hesseling AC, Rabie H, Marais BJ (2006). Bacille Calmette-Guérin vaccine-induced disease in HIV-infected and HIV-uninfected children. Clin Infect Dis.

[6] Fink JK, Squire LR (2004). Niemann–Pick Disease. Encyclopedia of Neuroscience.

[7] von Ranke FM, Pereira Freitas HM, Mançano AD (2016). Pulmonary Involvement in Niemann–Pick Disease: A State-of-the-Art Review. Lung.

[8] Vanier MT (2010). Niemann-Pick disease type C. Orphanet Journal of Rare Diseases.

[9] Yu X, Li X, Zhao G (2012). OxLDL up-regulates Niemann-Pick type C1 expression through ERK1/2/COX-2/PPAR -signaling pathway in macrophages. Acta Biochim Biophys Sin.

[10] Platt N, Speak AO, Colaco A (2016). Immune dysfunction in Niemann-Pick disease type C. J Neurochem.

[11] Liu CH, Liu H, Ge B (2017). Innate immunity in tuberculosis: host defense vs pathogen evasion. Cell Mol Immunol.

[12] Vrieling F, Wilson L, Rensen PCN, Walzl G, Ottenhoff THM, Joosten SA (2019). Oxidized low-density lipoprotein (oxLDL) supports Mycobacterium tuberculosis survival in macrophages by inducing lysosomal dysfunction. PLOS Pathogens.

[13] Wheelwright M, Kim EW, Inkeles MS (2014). All-Trans Retinoic Acid-Triggered Antimicrobial Activity against Mycobacterium tuberculosis Is Dependent on NPC2. J Immunol.

[14] Palanisamy GS, Kirk NM, Ackart DF (2012). Uptake and accumulation of oxidized low-density lipoprotein during Mycobacterium tuberculosis infection in guinea pigs. PloS one.

[15] Pandey P, Bhatnagar AK, Mohan A (2019). Insights in tuberculosis immunology: Role of NKT and T regulatory cells. Int J Mycobacteriol.

[16] Rothchild AC, Jayaraman P, Nunes-Alves C, Behar SM (2014). iNKT cell production of GM-CSF controls Mycobacterium tuberculosis. PLoS Pathog.

[17] Fineran P, Lloyd-Evans E, Lack NA (2016). Pathogenic mycobacteria achieve cellular persistence by inhibiting the Niemann-Pick Type C disease cellular pathway. Wellcome Open Res.

[18] Schwerd T, Pandey S, Yang HT (2017). Impaired antibacterial autophagy links granulomatous intestinal inflammation in Niemann-Pick disease type C1 and XIAP deficiency with NOD2 variants in Crohn’s disease. Gut.

